#  Dedifferentiated Peripheral Chondrosarcoma: A Review of Radiologic Characteristics

**DOI:** 10.1155/2013/505321

**Published:** 2013-03-25

**Authors:** Eric R. Henderson, Elisa Pala, Andrea Angelini, Eugenio Rimondi, Pietro Ruggieri

**Affiliations:** ^1^Orthopaedic Oncology, Dartmouth-Hitchcock Medical Center, The Geisel School of Medicine at Dartmouth, Lebanon, NH 03756, USA; ^2^Istituto Ortopedico Rizzoli, 40136 Bologna, Italy; ^3^University of Bologna, 40126 Bologna, Italy

## Abstract

*Introduction.* Peripheral de-differentiated chondrosarcomas are among the rarest malignant mesenchymal tumors. This tumor's descriptive radiographic characteristics are reported but objective quantification does not exist. This investigation surveyed imaging of peripheral de-differentiated chondrosarcomas to facilitate better recognition of these uncommon tumors. *Methods.* Database interrogation for peripheral de-differentiated chondrosarcomas was performed; 23 patients were identified and imaging for 18 was reviewed. A musculoskeletal radiologist reviewed all studies for mineralization characteristics; presence of pre-existing osteochondromas; preserved corticomedullary continuity; adjacent cortical obliteration; soft-tissue mass; tumor necrosis; and presence of a cartilage cap. Tumor luminance was measured with computer software. *Results.* Mineralization was present in 17 tumors. Pre-existing exostoses were evident in nine cases, corticomedullary continuity was preserved in three cases. There was no difference in mineralization or other characteristics based on tumor location. Mean tumor luminance was 94.9 candela/m^2^. *Conclusions.* The imaging characteristics described for central de-differentiated chondrosarcomas are similar to the peripheral form of this tumor. Peripheral mineralization with a bimorphic pattern on CT scan and the presence of a soft-tissue mass should be considered worrisome for a peripheral de-differentiated chondrosarcoma, particularly in the setting of multiple hereditary exostoses.

## 1. Introduction

 Dedifferentiated chondrosarcoma is an uncommon tumor that is known to arise from preexisting, low-grade cartilage lesions [1–5]. This tumor demonstrates bimorphic histology with a well-differentiated cartilaginous component and a dedifferentiated, noncartilaginous component [4]. These lesions comprise approximately 11% of chondrosarcomas and generally occur in association with a central chondrosarcoma [3]. Because of its cartilaginous origin, dedifferentiated chondrosarcoma may also occur in the setting of a preexisting exostosis; however, the occurrence is rare [6]. When single-institution, redundant reporting is considered, approximately 60 discrete cases of peripheral dedifferentiated chondrosarcoma have been reported in limited series with an emphasis on descriptive reporting of histologic subtypes and patient survival [3, 4, 6–18]. 

 A consistent radiographic appearance of conventional, central dedifferentiated chondrosarcoma is recognized and described [19]; as a result radiologists and surgeons trained in musculoskeletal imaging are able to identify these lesions successfully. The typical radiographic description of a central dedifferentiated chondrosarcoma is a lesion that originates within bone with an area of cortical breach and subsequent soft-tissue mass demonstrating a bimorphic pattern with mineralized and unmineralized areas; pathologic fracture is common [19]. Unlike the more common, central lesions, peripheral dedifferentiated chondrosarcomas arise from preexisting exostoses or, extracortically, and may appear as a peripheral chondrosarcoma without the features of its dedifferentiated counterpart [19].

 Descriptive reporting of radiologic findings has been undertaken in some case reports and limited series; however, objective quantification of radiographic characteristics for peripheral dedifferentiated chondrosarcoma has not been performed. The varied descriptions of this tumor have included comparisons to a normal osteochondroma, a low-grade chondrosarcoma, and a conventional dedifferentiated chondrosarcoma [19, 20]. The purposes of this investigation were to quantify and describe the radiographic findings of a large series of patients with peripheral dedifferentiated chondrosarcomas to determine whether this tumor has a distinct, recognizable radiographic appearance. 

## 2. Patients and Methods

 After Investigational Review Board and Ethics Committee approvals were obtained, the senior investigator's institutional database was queried for patients treated between 1980 and 2012 with a diagnosis of peripheral dedifferentiated chondrosarcoma; 23 patients were identified. Five patients were encountered only in consultation, and no imaging studies were available, leaving 18 patients for consideration. 

 The mean age of the patients at the time of operation was 46.4 years (range from 22.9 to 70.0 years). There were 13 men and five women. Nine patients' tumors arose from an exostosis in the setting of multiple hereditary exostoses (MHE), eight lesions arose from a preexisting solitary exostosis, and one lesion arose peripherally without an exostosis. The cartilaginous component of the tumors was chondrosarcoma in all patients. The histologic subtype of the dedifferentiated, noncartilaginous component was malignant fibrous histiocytoma-like (MFH) in 11 patients, osteosarcoma-like in five patients, and spindle-cell sarcoma-like in two patients. There was one lesion of the sternum, two of the scapula, three of the humerus, five of the pelvis, four of the femur, two of the tibia, and one of the fibula. 

 Preoperative imaging studies of the lesion included plain radiographs alone for five patients; a computed tomography (CT) scan alone for one patient; plain radiographs and CT for eight patients; and plain radiographs, CT, and magnetic resonance imaging (MRI) for four patients. An attending-level musculoskeletal radiologist evaluated all imaging studies. Plain radiographs were assessed for the presence of mineralization, whether mineralization appeared to encompass greater or less than 50% of the tumor area, a bimorphic pattern of mineralization, the presence of a soft-tissue mass, evidence of a preexisting exostosis, evidence of preserved corticomedullary continuity when an exostosis was present, extracompartmental extension, and erosion of the adjacent cortex. CT and MRI scans were assessed for the presence of mineralization, whether mineralization was central, peripheral, or both; a bimorphic pattern of mineralization; the presence of a soft-tissue mass; evidence of a preexisting exostosis; preserved corticomedullary continuity; extracompartmental extension; erosion of the adjacent cortex; the presence and thickness of a cartilage cap, and necrosis. 

Tumor luminance was measured in an effort to objectively quantify tumor mineralization content on plain radiographs. Luminance is a measurement of brightness with units of candela per square meter; it is measured on a scale of zero, or completely black, to 255, or completely white. Luminance has been used in previous investigations to measure trabecular and soft-tissue density with both radiography and ultrasound [21, 22]. Radiographs intended for analysis were displayed on a conventional light box (Dupix, Milano, Italy) and photographed with a 12-megapixel digital camera at 50 cm range (Canon A1100IS, Canon USA, Lake Success, NY, USA). Digital images were saved as Joint Photographic Experts Group (JPEG) files without compression and were opened with GNU Image Manipulation Program version 2.8 (GIMP Developers, Groton, MA, USA). The manual selection tool was used to trace the periphery of the tumor, and the histogram function was used to measure the mean luminance of the tumor. The selection was then inverted to measure the mean luminance of the surrounding soft tissues. Luminance of the soft tissues was subtracted from tumor luminance to yield a measurement of net tumor luminance. These numbers were recorded in a spreadsheet (Microsoft Excel for Mac 2011, Microsoft Inc., Redmond, WA, USA). Means were compared statistically with the Student's *t* test.

## 3. Results

 Plain radiographs revealed mineralization was seen in 16 of 17 cases; in eight cases it appeared to be bimorphic (Table 1). Mineralization appeared to occupy more than half of the tumor area in 10 patients and less than half in six patients. A soft-tissue mass was seen in 14 of 17 cases. Preexisting exostoses at the tumors' origin could be identified in eight of 17 cases; corticomedullary continuity appeared to be preserved in three cases. Thirteen cases showed evidence of adjacent cortical erosion. No patients had a pathologic fracture. 

 Dedifferentiated, noncartilaginous histologic subtypes of patients in this series included 11 with MFH-like components, five with osteosarcoma-like components, and two patients with a spindle-cell sarcoma-like components. Mineralization was seen in 10 of 11 patients with MFH-like tumors, and in all patients with osteosarcoma-like and spindle-cell-like tumors, there was no statistical difference between groups (Table 1). 

 There were eight axial lesions, seven of which demonstrated mineralization. All appendicular lesions showed mineralization. There was no difference in the occurrence of mineralization when results were divided by tumor location (Table 2). 

Computed tomography demonstrated mineralization in 12 of 13 scans, it was thought to be bimorphic in 11 cases. The only CT to not demonstrate mineralization was the case which did not show mineralization on plain radiographs. Cross-sectional imaging showed the mineralization to be peripheral-only in four cases and central and peripheral in eight cases; there were no cases of central-only mineralization. A soft-tissue mass was identified in all 13 CT scans. A preexisting exostosis was seen in six CT scans; four of these exostoses were identified on the corresponding plain radiographs, and two were not identified on plain radiographs. Corticomedullary continuity was preserved in three cases with identifiable exostoses. Central necrosis was identified in three CT and MRI studies performed with contrast. A cartilage cap was identified in three lesions. There was no difference when results were divided by histologic subtype or tumor location (Tables 3 and 4). 

 Tumor luminance was measured on plain radiographs in 14 cases. Mean tumor luminance without soft-tissue subtraction was 138.7 candela/m^2^ (range, 70.5 to 201.2). Mean adjacent soft-tissue luminance was 43.8 candela/m^2^ (range from 11.1 to 140.0); therefore mean luminance for the tumors alone was 94.9 candela/m^2^ (range from 59.4 to 129.0), indicating that tumor opacity on radiographs was approximately 37%. When compared by histological subtype, MFH-like and OSA-like tumor luminance showed no statistical difference (*P* = 0.49). When spindle cell-like tumors were compared to MFH-like and OSA-like, the difference approached significance (*P* = 0.14), however, limited patient numbers precluded robust statistical analysis (Table 1).

## 4. Discussion

**Figure 1:**
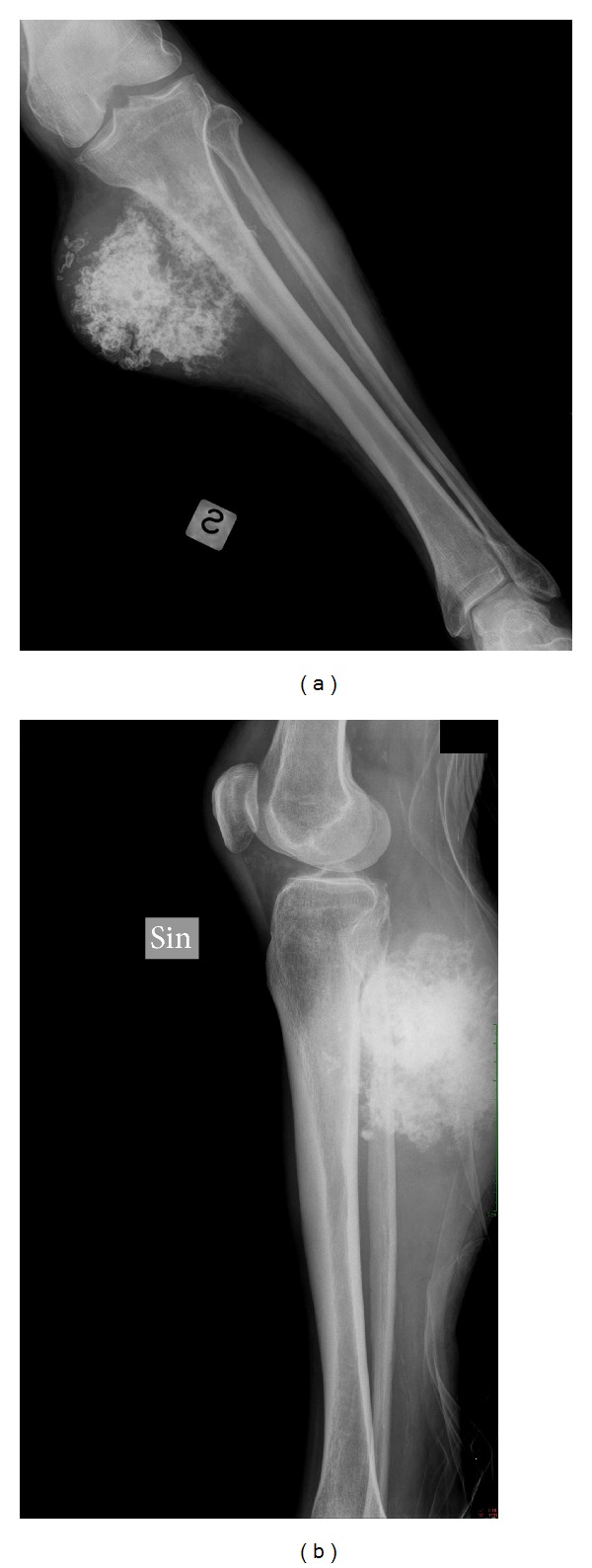
(a) AP and (b) lateral radiographs of tibia and fibula demonstrating a peripheral dedifferentiated chondrosarcoma with mineralization and a soft-tissue mass.

**Figure 2:**
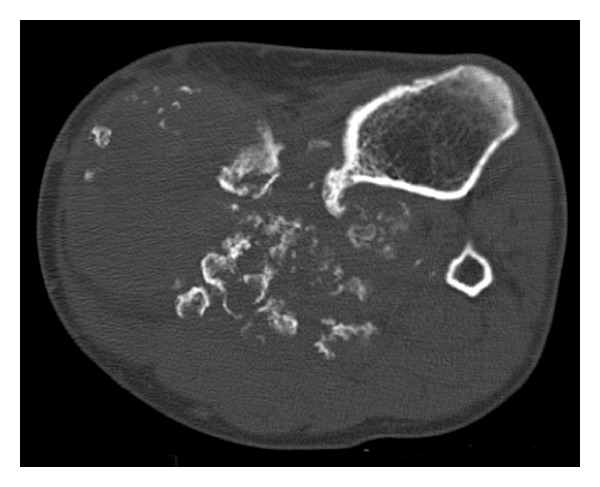
Axial CT scan of tibia and fibula demonstrating a peripheral dedifferentiated chondrosarcoma with central and peripheral, bimorphic mineralization, destruction of the prior exostosis, and a soft-tissue mass.

**Figure 3:**
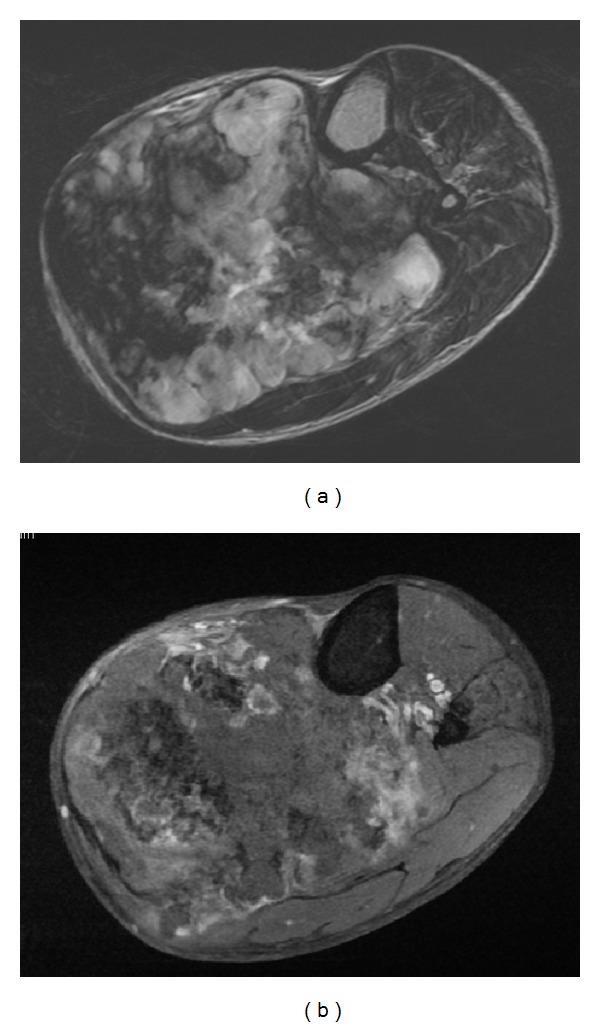
Axial MRI scan of tibia and fibula demonstrating a peripheral dedifferentiated chondrosarcoma with soft-tissue mass and heterogeneous T2 weighted (a) and T1 weighted with contrast (b) enhancement.

 Anderson and coauthors reported the earliest description of a peripheral dedifferentiated chondrosarcoma and noted that its radiographic characteristics were consistent with osteochondroma [20]. Since that time small series of peripheral dedifferentiated chondrosarcomas have been published with descriptive accounts of this tumor's radiographic appearance but without objective quantification of findings. The present study demonstrates that mineralization is present in the majority of peripheral dedifferentiated chondrosarcomas (Figure 1). Mineralization patterns, best visualized with CT scan, are usually central and peripheral or peripheral-only (Figure 2). A bimorphic mineralization pattern was demonstrated more reliably with CT than plain radiographs; however, it cannot be relied upon as a definite indicator of tumor de-differentiation. Obliterations of the preexisting exostosis or adjacent cortex are common findings; however, the presence of a soft-tissue mass on CT or MRI was the most consistent radiographic feature associated with peripheral dedifferentiated chondrosarcomas (Figure 3). 

 The radiographic characteristics of conventional, central chondrosarcomas are known and include deep endosteal scalloping, cortical destruction, and a soft-tissue mass [23]. Garrison and coauthors were the first to describe a large series of secondary chondrosarcomas arising from osteochondromas. Radiographic features consistent with malignant degeneration included an indistinct superficial border, the presence of a partially mineralized soft-tissue mass, and frequent destruction of the underlying osteochondroma [13]. Wuisman and coauthors mentioned only blurring of the bone borders as an indicator of malignant transformation [24]. Ahmed and coauthors, in a series of 107 patients with secondary chondrosarcomas arising from exostoses, documented irregular margins, heterogeneous mineralization, and a soft-tissue mass as positive indicators of malignant change in an osteochondroma [25]. Altay and coauthors reported a series of 32 patients with malignant degeneration of an osteochondroma but did not comment on radiological features [26]. 

 Mercuri and coauthors reported that the imaging characteristics of central dedifferentiated chondrosarcoma depended on the preexisting cartilage tumor [19]. They noted that when the noncartilaginous component was small, the imaging findings often reflected a conventional chondrosarcoma. When the dedifferentiated, noncartilaginous component was larger, however, the tumor often demonstrated no discernible radiographic characteristics of a cartilaginous neoplasm. The two features they found most commonly on plain radiographs were permeative osteolysis and a soft-tissue mass. The largest series to address radiographic features of central dedifferentiated chondrosarcomas was published by Littrell and coauthors [16]. The authors reported cortical destruction, chondroid matrix, soft-tissue mass, and tumor bimorphism were the most common findings associated with these tumors; as in the present study, CT was more sensitive in detecting the mineralized component as well as demonstrating bimorphism. Radiographic characteristics described by Johnson and coauthors included a lytic lesion with cortical destruction and a soft-tissue mass [27].

Several case series of dedifferentiated chondrosarcoma have included mixed reporting of central and peripheral tumors. Discrete accounts of radiologic features for peripheral dedifferentiated chondrosarcoma include few case reports and limited case series. Cortical destruction was the first described harbinger of de-differentiation [17]. Bertoni and coauthors published the earliest series of peripheral dedifferentiated chondrosarcomas and described a preexisting osteochondroma, cortical destruction, and a soft-tissue mass as consistent findings among all tumors [6]. Staals and coauthors described the largest series of peripheral dedifferentiated chondrosarcomas. Radiographs of all patients demonstrated indistinct borders, heterogeneous mineralization, and a soft-tissue mass [7]. Bimorphic mineralization was noted in half of their patients and, similar to the current study, was more evident with CT than plain radiographs. 

The current investigation confirms that the primary radiologic features of central dedifferentiated chondrosarcoma, soft-tissue mass, heterogeneous mineralization, and bimorphism are similar to the less common peripheral lesion morphology (Figure 1). Secondary features of central tumors including intramedullary mineralization with an extramedullary, radiolucent soft-tissue mass and pathologic fracture [19] are uncommon with peripheral lesions, likely due to the origin of the lesion outside of the medullary space. 

Over one-half of the cases in the current series showed an MFH-like histologic morphology. This finding diverges from the largest reports of central dedifferentiated chondrosarcoma where MFH-like features comprised from 4% to 22% of the total cases, and osteosarcoma-like characteristics usually dominate [2, 3, 16, 28]. Other reports have documented a rate of MFH-like histologic subtypes greater than 50% in the peripheral form of dedifferentiated chondrosarcoma, indicating that the peripheral form may have a predilection for this morphology [7, 12, 15]. 

This investigation has limitations that warrant discussion. Our study details the radiologic findings of eighteen patients treated over a 33-year interval, during which imaging technology changed substantially, creating a heterogeneous mix of radiographic studies. While our case number is small and underpowered to truly ascertain statistical differences in the radiographic appearances of these rare lesions, this study represents the largest and only investigation dedicated to peripheral dedifferentiated chondrosarcoma imaging, and we believe that the results justify reporting. Measurement of tumor luminance in this investigation was an attempt to quantify tumor opacity, and therefore its mineralized content is relative to the surrounding soft tissues. Further investigations correlating this technique with quantitative CT are required to determine its usefulness and validate the results; however, the authors believe that it may prove a useful technique for quantitifying mineralization in the absence of advanced, three-dimensional imaging. The authors acknowledge that luminance in isolation does not provide radiologists and surgeons with objective criteria for ruling peripheral dedifferentiated chondrosarcoma in or out as a diagnosis; however, it does provide an objective starting point for comparison to other tumors that could lead to such parameters. 

## 5. Conclusion

 In general the imaging characteristics described for central dedifferentiated chondrosarcomas are applicable to the peripheral form of this tumor. Peripheral mineralization with a bimorphic pattern on CT scan and the presence of a soft-tissue mass should be considered worrisome for a peripheral dedifferentiated chondrosarcoma, particularly in the setting of multiple hereditary exostoses. 

## Figures and Tables

**Table 1:** Plain radiograph findings by histologic subtype.

	Mineralization				Soft-tissue mass			Exostosis		Tumor luminance
	Present	<50%	>50%	Bimorphic	Present	Extracompartmental	Cortical obliteration	Present	Corticomedullary continuity	
MFH-like subtype	9	2	7	4	10	10	8	7	1	103.9
OSA-like subtype	5	3	2	3	5	5	4	3	2	90.4
SCS-like subtype	2	1	1	1	2	2	1	1	0	67.6

Abbreviations: malignant fibrous histiocytoma (MFH); osteosarcoma (OSA); spindle-cell sarcoma (SCS).

**Table 2:** Plain radiograph findings by tumor location.

	Mineralization				Soft-tissue mass			Exostosis		Tumor luminance
	Present	<50%	>50%	Bimorphic	Present	Extracompartmental	Cortical obliteration	Present	Corticomedullary continuity	
Axial	6	2	4	5	7	7	8	5	2	93.2
Appendicular	10	4	6	3	10	10	5	6	1	95.8

Abbreviations: malignant fibrous histiocytoma (MFH); osteosarcoma (OSA); spindle-cell sarcoma (SCS).

**Table 3:** CT findings by histologic subtype.

	Mineralization				Soft-tissue mass				Exostosis		
	Present	Peripheral	Central	Bimorphic	Present	Extracompartmental	Cortical obliteration	Necrosis	Present	Corticomedullary continuity	Cartilage cap
MFH-like subtype	6	6	2	6	7	7	7	2	2	1	1
OSA-like subtype	4	4	2	3	4	4	3	1	3	2	1
SCS-like subtype	2	2	2	2	2	2	1	0	1	0	1

Abbreviations: malignant fibrous histiocytoma (MFH); osteosarcoma (OSA); spindle-cell sarcoma (SCS).

**Table 4:** CT findings by tumor location.

	Mineralization				Soft-tissue mass				Exostosis		
	Present	Peripheral	Central	Bimorphic	Present	Extracompartmental	Cortical obliteration	Necrosis	Present	Corticomedullary continuity	Cartilage cap
Axial	6	6	5	5	7	7	7	2	2	2	2
Appen- dicular	6	6	3	6	6	6	4	1	4	1	1

## References

[1] Dickey ID, Rose PS, Fuchs B (2004). De-differentiated chondrosarcoma: the role of chemotherapy with updated outcomes. *The Journal of Bone & Joint Surgery*.

[2] Staals EL, Bacchini P, Bertoni F (2006). De-differentiated central chondrosarcoma. *Cancer*.

[3] Frassica FJ, Unni KK, Beabout JW, Sim FH (1986). De-differentiated chondrosarcoma. A report of the clinicopathological features and treatment of seventy-eight cases. *Journal of Bone and Joint Surgery, American Volume*.

[4] Dahlin DC, Beabout JW (1971). De-differentiation of low-grade chondrosarcomas. *Cancer*.

[5] Capanna R, Bertoni F, Bettelli G (1988). De-differentiated chondrosarcoma. *Journal of Bone and Joint Surgery, American Volume*.

[6] Bertoni F, Present D, Bacchini P (1989). De-differentiated peripheral chondrosarcomas. A report of seven cases. *Cancer*.

[7] Staals EL, Bacchini P, Mercuri M, Bertoni F (2007). De-differentiated chondrosarcomas arising in preexisting osteochondromas. *Journal of Bone and Joint Surgery, American Volume*.

[8] Park YK, Yang MH, Ryu KN, Chung DW (1995). De-differentiated chondrosarcoma arising in an osteochondroma. *Skeletal Radiology*.

[9] Astorino RN, Tesluk H (1985). De-differentiated chondrosarcoma with a rhabdomyosarcomatous component. *Human Pathology*.

[10] Bennett GE, Berkheimer GA (1941). Malignant degeneration in a case of multiple benign exostoses with a brief review of the literature. *Surgery*.

[11] Dekker AP, Grimer RJ (2012). Transformation of solitary osteochondroma to de-differentiated chondrosarcoma arising in the distal radius: a case report. *Musculoskeletal Surgery*.

[12] Franchi A, Baroni G, Sardi I, Giunti L, Capanna R, Campanacci D (2012). De-differentiated peripheral chondrosarcoma: a clinicopathologic, immunohistochemical, and molecular analysis of four cases. *Virchows Archiv *.

[13] Garrison RC, Unni KK, McLeod RA (1982). Chondrosarcoma arising in osteochondroma. *Cancer*.

[14] Grimer RJ, Karpinski MRK, Edwards AN (1984). The long-term results of Stanmore total knee replacements. *Journal of Bone and Joint Surgery, British Volume*.

[15] Kilpatrick SE, Pike EJ, Ward WG, Pope TL (1997). De-differentiated chondrosarcoma in patients with multiple osteochondromatosis: report of a case and review of the literature. *Skeletal Radiology*.

[16] Littrell LA, Wenger DE, Wold LE (2004). Radiographic, CT, and MR imaging features of De-differentiated chondrosarcomas: a retrospective review of 174 De Novo cases. *Radiographics*.

[17] Sissons HA (1979). Case report 83. *Skeletal Radiology*.

[18] Voutsinas SA (1988). Spindle-cell sarcoma in patients who have osteochondromatosis. A report of two cases. *Journal of Bone and Joint Surgery, American Volume*.

[19] Mercuri M, Campanacci L (1995). De-differentiated chondrosarcoma. *Skeletal Radiology*.

[20] Anderson RL, Popowitz L, Li JK (1969). An unusual sarcoma arising in a solitary osteochondroma. *Journal of Bone and Joint Surgery, American Volume*.

[21] Rafferty KL, Ruff CB (1994). Articular structure and function in Hylobates, Colobus, and Papio. *American Journal of Physical Anthropology*.

[22] Wu JS, Darras BT, Rutkove SB (2010). Assessing spinal muscular atrophy with quantitative ultrasound. *Neurology*.

[23] Murphey MD, Flemming DJ, Boyea SR, Bojescul JA, Sweet DE, Temple HT (1998). Enchondroma versus chondrosarcoma in the appendicular skeleton: differentiating features. *Radiographics*.

[24] Wuisman PIJM, Jutte PC, Ozaki T (1997). Secondary chondrosarcoma in osteochondromas. Medullary extension in 15 of 45 cases. *Acta Orthopaedica Scandinavica*.

[25] Ahmed AR, Tan TS, Unni KK, Collins MS, Wenger DE, Sim FH (2003). Secondary chondrosarcoma in osteochondroma: report of 107 patients. *Clinical Orthopaedics and Related Research*.

[26] Altay M, Bayrakci K, Yildiz Y, Erekul S, Saglik Y (2007). Secondary chondrosarcoma in cartilage bone tumors: report of 32 patients. *Journal of Orthopaedic Science*.

[27] Johnson S, Têtu B, Ayala AG, Chawla SP (1986). Chondrosarcoma with additional mesenchymal component (De-differentiated chondrosarcoma): I. A clinicopathologic study of 26 cases. *Cancer*.

[28] Grimer RJ, Gosheger G, Taminiau A (2007). De-differentiated chondrosarcoma: prognostic factors and outcome from a European group. *European Journal of Cancer*.

